# Selective Coupling between Theta Phase and Neocortical Fast Gamma Oscillations during REM-Sleep in Mice

**DOI:** 10.1371/journal.pone.0028489

**Published:** 2011-12-05

**Authors:** Claudia Scheffzük, Valeriy I. Kukushka, Alexei L. Vyssotski, Andreas Draguhn, Adriano B. L. Tort, Jurij Brankačk

**Affiliations:** 1 Institute for Physiology and Pathophysiology, University Heidelberg, Heidelberg, Germany; 2 Quantum Chromoplasma Laboratory, Dniepropetrovsk National University, Dniepropetrovsk, Ukraina; 3 Institute of Neuroinformatics, University of Zürich/ETH, Zürich, Switzerland; 4 Brain Institute, Federal University of Rio Grande do Norte, Natal, Rio Grande do Norte, Brazil; The Research Center of Neurobiology-Neurophysiology of Marseille, France

## Abstract

**Background:**

The mammalian brain expresses a wide range of state-dependent network oscillations which vary in frequency and spatial extension. Such rhythms can entrain multiple neurons into coherent patterns of activity, consistent with a role in behaviour, cognition and memory formation. Recent evidence suggests that locally generated fast network oscillations can be systematically aligned to long-range slow oscillations. It is likely that such cross-frequency coupling supports specific tasks including behavioural choice and working memory.

**Principal Findings:**

We analyzed temporal coupling between high-frequency oscillations and EEG theta activity (4–12 Hz) in recordings from mouse parietal neocortex. Theta was exclusively present during active wakefulness and REM-sleep. Fast oscillations occurred in two separate frequency bands: gamma (40–100 Hz) and fast gamma (120–160 Hz). Theta, gamma and fast gamma were more prominent during active wakefulness as compared to REM-sleep. Coupling between theta and the two types of fast oscillations, however, was more pronounced during REM-sleep. This state-dependent cross-frequency coupling was particularly strong for theta-fast gamma interaction which increased 9-fold during REM as compared to active wakefulness. Theta-gamma coupling increased only by 1.5-fold.

**Significance:**

State-dependent cross-frequency-coupling provides a new functional characteristic of REM-sleep and establishes a unique property of neocortical fast gamma oscillations. Interactions between defined patterns of slow and fast network oscillations may serve selective functions in sleep-dependent information processing.

## Introduction

Most neuronal activity is entrained by local or global network oscillations. Early observations of EEG patterns have already revealed the state-dependence of different oscillations [1]. Meanwhile, multiple lines of evidence support selective roles of different rhythmic activity patterns for different cognitive and behavioural tasks including memory formation, memory consolidation, attention, perception, action preparation, locomotion and others [2]–[6]. Different oscillatory states extend over different distances, with a general tendency of high frequencies synchronizing activity within short distances and low frequencies mediating long-range coherence [7]–[10]. However, highly distributed patterns of activity require that fast oscillations are also synchronized over large distances [11]–[12]. The cellular processes underlying such fast long-range synchrony are far from understood. One candidate mechanism may be temporal coupling between local, high-frequency network oscillations and long-range oscillations at lower frequencies [7]–[9], [13]–[14]. Indeed, behavioural studies in animals and humans indicate that cross-frequency coupling (CFC) is involved in specific cognitive tasks [15], including behavioural choice [16] and working memory [17].

Coupling between theta (4 – 12 Hz) and gamma (30–100 Hz) oscillations has recently been observed in the rodent parietal neocortex [14]. In rodents, theta oscillations are generated within the hippocampal network and are volume-conducted to the neocortex [18], different from neocortical theta in humans [19], [20]. Here, we will use the term “EEG theta” to describe volume-conducted theta of hippocampal origin in our recordings from rodents. In contrast, fast oscillations in the gamma frequency range are organized by local mechanisms [10], [21]. The coupling between theta and gamma indicates specific interactions between the hippocampus and the neocortex, suggestive of a role in memory consolidation [22]. Oscillations of higher frequencies (>100 Hz) have been described in different species and locations. In humans and primates, high frequency oscillations in the neocortex (up to 500 Hz) are frequently characterized as “high gamma” [23]–[25]. In the rodent hippocampus, brief episodes of high frequency oscillations (>100 Hz) form a well-characterized network pattern termed ripples [10], [22]. Ripples are typically superimposed on propagating sharp waves, forming sharp wave-ripple complexes (SPW-R). They occur predominantly in non-theta states, i.e. quiet wakefulness and NREM-sleep [10], [26]–[29]. In the rodent neocortex, there are also high-frequency oscillations (>100 Hz). However, this pattern occurs during theta activity and is termed “neocortical gamma” [14]. “Neocortical ripples” in cats, again, occur during slow-wave sleep [30]. Together, fast neocortical network oscillations are generated locally [11], [31], [32] and are linked to specific vigilance states. Thus, they mark a distinct type of network activity.

In the present work, we have analyzed temporal relationships between fast oscillations in the parietal neocortex and EEG theta of freely moving mice. We found two different frequency domains of fast oscillations which are coupled to the underlying slow theta activity: gamma (40–100 Hz) and fast gamma oscillations (120–160 Hz). Importantly, cross-frequency coupling (CFC) was state-dependent with strongest theta-fast gamma CFC during REM-sleep. Our findings differentiate neocortical fast gamma from lower frequency (<100 Hz) gamma oscillations, and they demonstrate for the first time a strong dependence of CFC on vigilance state.

## Results

**Figure 1:**
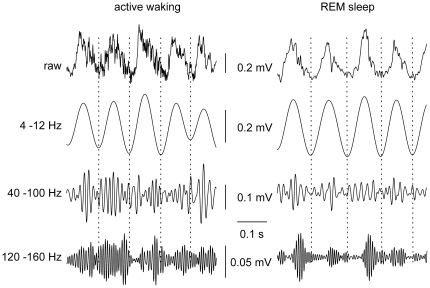
Raw signals during both theta states. Raw and filtered field potentials recorded from the parietal cortex during active waking and REM-sleep of one representative animal. Filtered traces emphasize theta range (second line, 4–12 Hz), gamma range (third line, 40–100 Hz), and fast gamma ranges (bottom, 120–160 Hz). Note higher fast gamma activity in active waking but more pronounced theta-phase coupling in REM-sleep.

**Figure 2:**
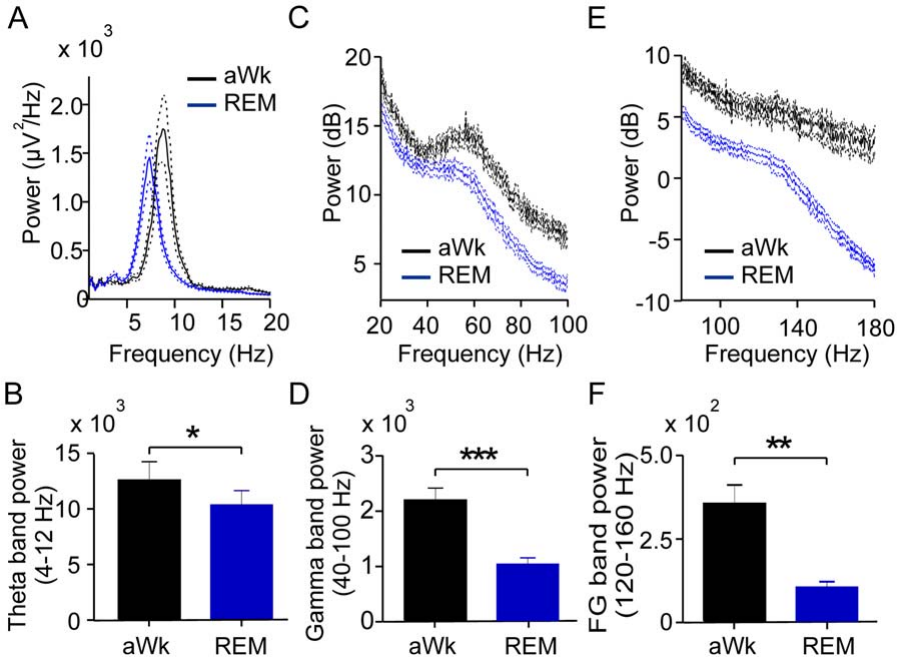
Power-frequency distributions and vigilance state. A, C, E: Mean power-frequency distributions emphasizing the theta, gamma and fast gamma ranges during active waking (aWk; black lines) and REM-sleep (REM; blue lines). Dotted lines represent SEM (n = 10 mice). Note the different power scales (dB) in C and E compared to A, B, D, F (µV^2^/Hz): Bar diagrams show mean band power for (B) theta (4–12 Hz), (D) gamma (40–100 Hz) and (F) fast gamma (FG, 120–160 Hz) during aWk (black columns) and REM blue columns). Asterisks in B, D, F indicate significant differences between active waking compared to REM-sleep (B, t-test, theta: p<0.05; D, gamma: p<0.0001; F, fast gamma: p<0.001).

**Figure 3:**
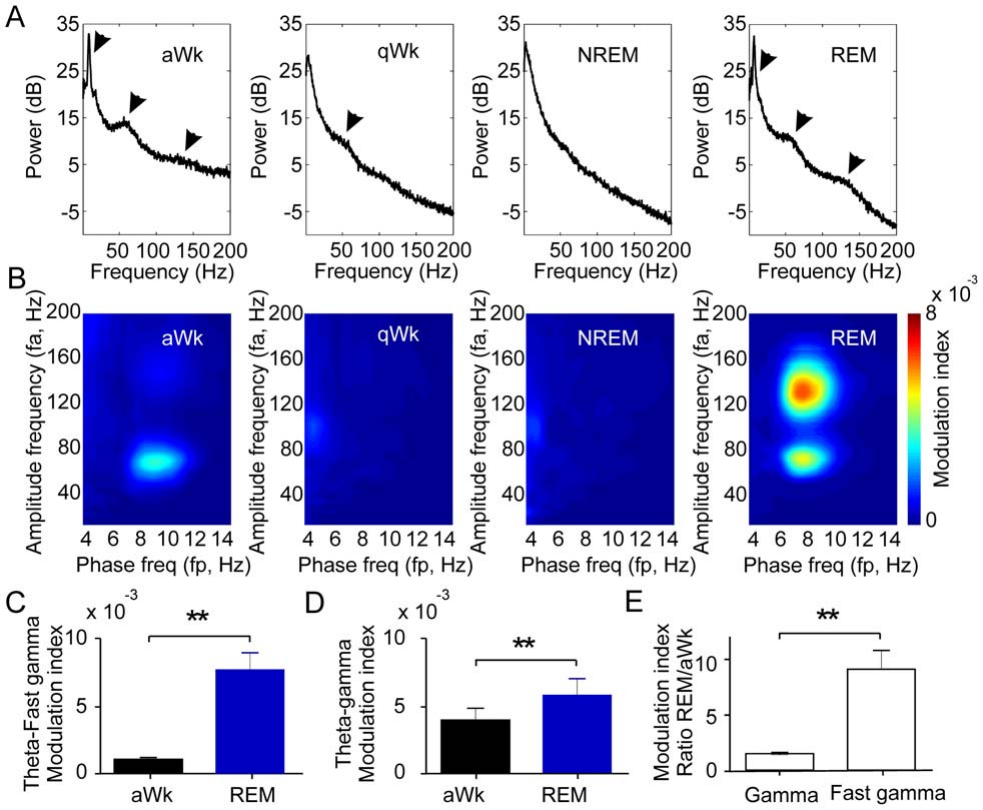
Power spectral densities (PSD) and cross-frequency coupling (CFC) in different vigilance states. A: PSDs during spontaneous active waking (aWk), quiet waking (qW), NREM- and REM-sleep (n = 10 mice). Arrows point to the power peaks for theta, gamma and fast gamma activity. B: Mean comodulation maps (CFC) in parietal cortex during the four behavioural states (n = 10 mice). The abscissa represents the phase-modulating frequencies (f_p_) and ordinate amplitude-modulated frequencies (f_a_). Pseudocolour scale indicates the modulation index (see Methods for details). C, D: Theta-gamma (C) and theta-fast gamma (D) CFCs are significantly higher in REM compared to aWk. E: Ratio of modulation indices in REM compared to aWk for gamma and fast gamma oscillations show significantly higher vigilance differences for theta-fast gamma compared to theta-gamma (p = 0.0014).

Field potentials were recorded from nineteen freely moving male mice for periods of three to ten hours using the cable-free neurologger system [33], [34]. As expected, waveform and spectral frequency content of cortical activity were clearly state-dependent with obvious differences between active wakefulness, resting immobility, slow-wave sleep and REM-sleep (for sleep staging see Methods and [35]). Prominent theta activity was exclusively present during active wakefulness and REM-sleep [36]. We therefore focused our analysis of coupling between theta and fast rhythmic activity to these two states (but see below for comparison with quiet waking and NREM-sleep). Visual inspection of raw data already revealed that theta waves were superimposed by different types of fast oscillations (Fig. 1 and Fig. S1A). This is in line with the known heterogeneity of high-frequency oscillations (>40 Hz) which are generated by a variety of mechanisms and serve different cognitive-behavioural tasks [16], [21], [25], [37]–[38]. Based on power spectrum analysis (Figs. 2C,E and 3A), we analyzed our recordings separately for neocortical gamma (40–100 Hz) and fast gamma oscillations (120–160 Hz).

All three frequencies (theta, gamma, fast gamma) had higher power during active wakefulness (aWk) as compared to REM-sleep (Fig. 2; n = 10). This difference was most pronounced for fast oscillations (gamma: 53%, fast gamma: 71%, Fig. 2D,F) while the mean theta band power (4–12 Hz) differed only by 12% (Fig. 2B). In addition, power-frequency plots revealed a faster peak frequency for theta in active wakefulness (∼9 Hz) as compared to REM-sleep (∼7 Hz). Similar peak frequencies for theta during tonic REM-sleep have been reported in rats [39]. Of note, while the three frequency bands were discernible in the spectral content of aWk and REM states, the quiet waking (qWk) state only exhibited a small peak in the gamma range whereas NREM had no visible high-frequency EEG component (Fig. 3A, n = 10).

Next, we searched for frequency coupling between theta and the two fast components (for details see Methods and [40]). As expected, cross-frequency coupling was exclusively present during aWk and REM (Fig. 3B), the two states with prominent theta- and high-frequency activity (Fig. 3A). Surprisingly, while power of all frequency bands was stronger in aWk as compared to REM (Fig. 2), coupling between the oscillations was clearly more pronounced during REM (Fig. 3B,C). Theta-fast gamma coupling was ∼9-fold stronger in REM-sleep as compared to active wakefulness (modulation index 0.0076±0.0013 versus 0.0010±0.0002; n = 10; p<0.005; Fig. 3C). State-dependence of theta-gamma coupling was less pronounced but also biased towards REM (modulation index 0.0058±0.0012 versus 0.0039±0.0009; n = 10; p<0.005; Fig. 3D). These data reveal that CFC is highly selective for different high-frequency bands and for different states of vigilance. The ratio between CFC in REM versus aWk for fast gamma was 9.0±1.7 times (range: 2.6–20.3 times) while theta-gamma coupling was only increased by a factor of 1.5±0.1 (range: 0.9-2.0 times; Fig. 3E; p = 0.0014; n = 10). The state-dependent and discontinuous distribution of modulation indices between gamma and fast gamma shows that these two frequency bands mark different network patterns.

**Figure 4:**
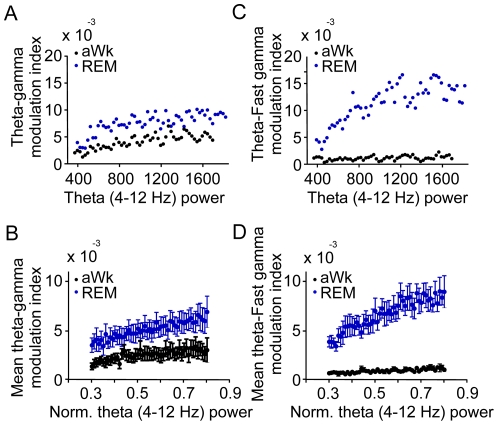
Theta phase coupling of gamma and fast gamma oscillations in parietal cortex depends on theta power, both in active waking (aWk) and REM-sleep (REM). A and C: Data from a representative animal. Gamma and fast gamma CFCs were calculated from 30s periods of REM and aWk for bins of 50 theta power units (µV^2^/Hz). B and D: After normalization of theta power (see Methods for details) 30s periods of aWk and REM were pooled for bins of 0.01 normalized theta power units and CFC for gamma and fast gamma calculated and averaged over animals (n = 8 mice). Note positive correlation in all four data sets (see Results). Power was calculated from 1s windows in steps of 0.5s.

In principle, the difference in coupling could be secondary to a state-dependent difference in absolute theta power such that episodes with strong theta show particularly strong CFC [23], [37]. In order to control for such effects, we computed mean theta power in epochs of 1s duration. Theta power was binned (see Methods), and epochs with same theta power level were pooled to a total duration of 30s for each animal and power bin. We then calculated CFC between theta and both types of high-frequency oscillations. Theta-gamma coupling was dependent on theta power for both, aWk and REM-sleep (Fig. 4A,B). The same was true for theta-fast gamma coupling (Fig. 4C,D). Regression analysis revealed significant correlations between theta power and the modulation index for both frequency bands (gamma: r^2^ = 0.08242, p<0.0001 for REM; r^2^ = 0.04765, p<0.0001 for aWk; fast gamma: r^2^ = 0.2065, p<0.0001 for REM; r^2^ = 0.08801 p<0.0001 for aWk; n = 10). Nevertheless, modulation indices were clearly higher during REM as compared to aWk (significant difference between both states for all values shown in Fig. 4B,D). As visible from Fig. 4C and D, theta-fast gamma coupling during REM-sleep was particularly strongly dependent on theta power, resulting in an increased difference against wakefulness at high theta power values. Together, these data exclude that the state-dependent differences in CFC are a direct effect of state-dependent differences in theta power.

A confounding factor in comparisons of CFC can be effects of waveform characteristics like high-frequency bursts on comodulation values. We therefore investigated whether a stronger “burst-like” behaviour of fast oscillations during REM could explain the increase in theta-fast gamma coupling. We applied three different methods to quantify the amplitude time-course of fast gamma and gamma oscillations in aWk and REM (see Text S1 and Figures S2 and S3). All applied algorithms revealed that fast gamma were more “bursty” than gamma oscillations, i.e. they deviated stronger from baseline activity. However, this difference was more pronounced in aWk than in REM (see Fig. S2), and hence cannot explain the selective increase in theta-fast gamma coupling during REM.

While theta activity in the rodent parietal neocortex is volume-conducted from the hippocampus [10], [14], [41], [42], high-frequency oscillations are likely generated by local neocortical networks [11], [14], [30]–[32]. As a control for this assumption we analyzed data from CA1 which had been recorded together with parietal EEG (see Fig. S4A). When high-frequency oscillations from the neocortical site were correlated with hippocampal theta, CFC was almost identical to the CFC within the neocortex. However, within CA1, there was almost no CFC between theta and fast gamma while theta-gamma coupling was prominent (see also [43]). These data are consistent with the known theta-gamma coupling within hippocampal networks [13], and support an independent cortical origin of fast gamma oscillations. To test this hypothesis further, cross-regional coherence analysis was performed between recordings from parietal cortex and two different electrode locations in CA1 (deep: Fig. S5A; stratum oriens: Fig. S5B). Coherence between neocortex and both hippocampal electrodes was highest for theta oscillations, consistent with volume conduction. In contrast, coherence was low for gamma and negligible for fast gamma, suggesting an independent origin of these oscillations.

**Figure 5:**
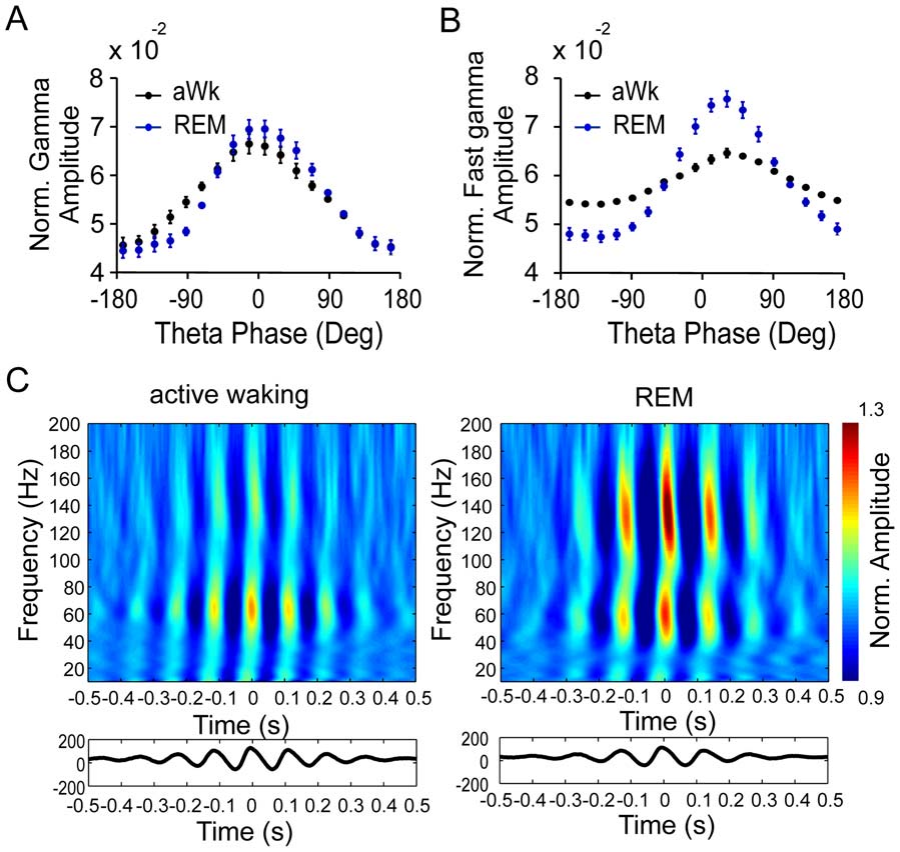
Gamma and fast gamma oscillations are differentially phase-locked to theta. A and B: Mean amplitudes of neocortical gamma (A, 40–100 Hz) and fast gamma (B, 120–160 Hz) plotted according to the phase of theta in active waking (black dots) and REM-sleep (blue dots). Amplitude maxima of gamma occur at the positive peak of the theta wave in parietal cortex (0 degrees), while the amplitude maxima of fast gamma occur at 30 degrees, on the falling flank of the positive peak of the theta wave. C: Time-frequency plot of mean amplitude distribution time-locked to the theta peak (n = 9 mice). The positive peaks of the averaged theta waves correspond to 0 s. Bottom panel shows the theta peak-locked averaged raw signal.

We next analyzed the phase-relationship between theta oscillations and the two high-frequency rhythms. Gamma amplitudes were highest at the positive peak of theta in our neocortical recordings (0°, Fig. 5A). This phase-dependence was not different between both vigilance states. Fast gamma oscillations, on the other hand, were most pronounced at ∼30° phase angle, corresponding to the decaying phase after the positive peak of the theta cycle (Fig. 5B). While the stronger coupling between theta and fast gamma in REM versus wakefulness is apparent from Fig. 5B, the phase-relationship was similar in both states. Phase-energy plots show the selective coupling of gamma and fast gamma activity to theta phase when the full high-frequency bandwidth is examined (Fig. 5C). These data confirm that both frequency components are strongly theta-modulated but reflect distinct network patterns.

Coupling between different oscillations has been suggested to serve specific cognitive functions, especially with respect to long-range synchronization and memory-related neuronal plasticity [2]–[3], [6], [11]-[12], [44]–[48]. We therefore tested whether exposure to a novel experience would alter the state-dependence of CFC. Nine additional mice were placed into a novel open field (NOF, Fig. S6A,B) and into an elevated plus maze (EPM, Fig. S6C,D for details see Methods). Cortical activity during active waking was compared between the first 30s of exploring the new environment and spontaneous exploration in the home cage (Fig. S6A for NOF and C for EPM). REM-sleep was compared immediately prior to and immediately after the exposure ( for NOF and D for EPM). State-dependent cross-frequency coupling in this independent series of experiments was identical to our previous observations. However, CFC patterns within the group of newly recorded mice were not affected by the behavioural paradigms.

## Discussion

We report three major results from EEG recordings in the parietal neocortex of freely behaving mice: i) the amplitude of gamma (40–100 Hz) and fast gamma oscillations (120–160 Hz) is coupled to the phase of theta (4–12 Hz) oscillations; ii) theta-fast gamma coupling is strongly (9-fold) enhanced during REM-sleep as compared to active wakefulness; iii) theta-gamma coupling is weakly (1.5-fold) enhanced during REM. The selective theta phase modulation of neocortical fast gamma oscillations during REM provides a new physiological marker of this state and distinguishes fast gamma from slower gamma rhythms.

Many behavioural and cognitive processes involve synchronous network oscillations. These rhythmic changes in excitability are believed to organize the spatial-temporal activity patterns of distributed neuronal assemblies, to support synaptic plasticity, and to define different functional states, e.g. different levels of vigilance [10]. Several brain regions are able to generate simultaneous oscillations at different frequencies, opening the possibility of systematic relationships between two rhythms. Such cross-frequency coupling has recently gained interest due to its possible involvement in memory processing [44]. An important example is theta-gamma coupling in the rodent and human hippocampus and neocortex [37], [49]–[51].

Different lines of evidence suggest that the interaction between slow, global activation cycles (theta) and local, fast synchrony (gamma) supports the synchronous activation of spatially distributed neuronal assemblies. Such mechanisms have been suggested to underlie perceptual binding [31], [47] and top-down visual processing [52]. With respect to memory functions, cross-frequency coupling may promote synaptic plasticity [53]. Indeed, theta-gamma coupling is selectively enhanced during working memory-dependent tasks in rats [16], [37] and humans [17]. More recently, theta-gamma coupling has also been found in the rat hippocampus during memory retrieval, indicating a role in long-term memory [37]. Prominent theta oscillations are expressed in the hippocampus during active behaviour with translational motion [36] and during REM-sleep [54]. Moreover, phase-coherence of theta has been observed between the prefrontal cortex and the hippocampus in animals during a spatial memory task [55]. Hippocampal ripples (>120 Hz), on the other hand, are most prominent during slow-wave sleep in the absence of marked theta oscillations [26], [29]. These fast oscillations have been implicated in memory consolidation [56]–[57], suggesting that they induce synaptic plasticity in cortical regions. Interestingly, synaptic plasticity is strongly supported by theta oscillations [45], pointing towards a potential function for the strong theta-fast gamma coupling in the neocortex. Theta-coupled cortical fast gamma oscillations appear most prominently during REM-sleep. This state has been associated with processing of recently experienced events, newly acquired skills, and regulation of emotions (for review see: [46], [58]–[61]). The behavioural, cognitive or mnestic function of state-specific coupling between theta and fast gamma oscillations remains presently unknown. Our behavioural experiments did not reveal a prominent experience-dependent change in CFC, but more subtle effects at the level of single units cannot be excluded.

With respect to the underlying cellular mechanisms it is surprising that local oscillations in the parietal cortex are strongly modulated by theta oscillations. Theta rhythms are not generated in the neocortex itself. Rather, they emerge from the network formed by the hippocampus, septal nuclei and entorhinal cortex [18]. In rodents, the parietal neocortex is immediately adjacent to the dorsal hippocampus, and field potential theta oscillations in this region are a result of volume conduction from the hippocampus proper (i.e. CA1 and CA3) and the entorhinal cortex [14]. The lack of independent neocortical current sinks or sources during theta has been confirmed by field potential depth profiles [41]–[42]. In contrast, theta rhythms within CA1 show a gradual polarity change between stratum oriens and the hippocampal fissure, consistent with local generation of theta in this region [62]–[65]. Despite their remote origin, theta rhythms have a clear influence on local network oscillations in the parietal cortex (our data) and can entrain neocortical unit discharges [14]. Thus, volume conducted field effects may be relevant for complex and precisely timed neuronal activity patterns [66]–[67]. Indeed, transcranial application of slow rhythmic electrical fields in humans affects EEG and memory performance [68]–[69]. Alternatively, weak connections between hippocampus and neocortex may directly affect neuronal activity without causing major field potentials.

In summary, we report a new and highly selective interaction between theta oscillations and two different types of neocortical fast network oscillations. Further behavioural experiments in combination with electrophysiological recordings are needed to elucidate a possible cognitive impact of both interactions. Likewise, the underlying cellular mechanisms can only be untangled with additional experiments in vivo and in vitro.

## Materials and Methods

### Ethics statement

This study was carried out in accordance with the European Science Foundation Policy on the Use of Animals in Research [70], the U.S. National Institutes of Health Guide for the Care and Use of Laboratory Animals [71] and has been approved by the Governmental Supervisory Panel on Animal Experiments of Baden Württemberg at Karlsruhe (35-9185.81/G-30/08).

### Animal Care and Housing Conditions

Male C57/Bl6n mice at either 28 or 45 days age were purchased from Charles River (Sulzfeld, Germany). For a minimum of two weeks they were housed in groups of four to five inside a ventilated Scantainer (Scanbur BK A/S Denmark) on an inverted 12/12-h light/dark cycle with light on from 20:00 to 8:00 and free access to water and food. After electrode implantation, animals were housed individually throughout the experiment.

### Animal Preparation

Nineteen male C57/Bl6n mice (29–37 g, 90-150 days old) were anesthetized with isoflurane in medical oxygen (4% isoflurane for induction, 1.5–2.5% for maintenance, flow rate: 1 l per min). Anesthetized animals were placed in a stereotactic apparatus with a custom-made inhalation tube. For analgesia, 0.1 mg/kg of buprenorphin was injected subcutaneously prior to and 8h after surgery. After exposure of the skull bone, two stainless steel or gold plated brass watch screws were fixed permanently into the skull, one screw was used for recording and placed over the left lateral parietal association cortex (2 mm posterior of bregma, 1.5 mm lateral to the midline). A second screw over the cerebellum served as ground and reference electrode. In mice, the lateral parietal cortex overlays the dorsal hippocampus permitting reliable recording of the theta rhythm with comparable amplitudes between different animals. The impedance of the epidural screw electrode was 7.1 kOhm at 100 Hz (range: 6.8-7.6) and 3.0 kOhm at 1kHz (range: 3.0–3.1). Two pairs of varnish-insulated nichrome wires (100 µm, glued together) cut with an angle of 45° were implanted into the right hippocampal CA1 area for a different set of experiments. One electrode was usually at stratum oriens the deeper one below stratum pyramidale. Another pair of identical wires cut square was inserted into the neck muscle for EMG recording, one of which served as EMG reference electrode. Due to the limited number of recording channels (see below) EMG recording was unilateral.

### Electrophysiology and recording of behaviour

One week after surgery, continuous monopolar electroencephalographic (EEG) recording began with a 24 h session in a Phenotyper home cage (Noldus Information Technology, Wageningen, Netherlands) measuring 30 cm × 30 cm with free access to food and water. Recordings were performed by using a miniaturized data logger (Neurologger 2A), an advanced version of the neurologger previously described [33], [34] with one of the references (EEG) connected to ground. The input impedance of all the recording channels, including reference channels was larger than 30 MOhm. Four channels of EEG and EMG signals were bandpass filtered (1 to 700 Hz, -3 dB, attenuation -6 dB/octave) and digitized by an on-board A/D-converter (6400 Hz per channel) after amplification at x1000 (input range of ± 1 mV). Means of sequential groups of four samples were stored in the on-board 512 MB non-volatile memory at a rate of 1600 Hz. The dimensions of the neurologger were 23x15x13 mm and the total weight was 3.6 g with two batteries (Renata ZA 10; Itingen, Switzerland). This weight includs the neurologger itself (1.4 g), the add-on accelerometer/infrared synchronization board (0.4 g), batteries (0.6 g), optional battery holders as well as protective casing. The head implant (electrodes, wires, contacts and dental acrylic) had an additional weight of 0.7 g. In spite of this relative heavy burden (more than 10% of the body weight) the movement of the mice was not visibly altered (for details see [35]). At the end of the experiment, the data was downloaded from the data logger onto a personal computer for further analysis. The animal's behaviour was continuously recorded by the video tracking system Ethovision XT 7.1 (Noldus Information Technology, Wageningen, Netherlands).

### Exposure to novel environment

Nine additional animals were exposed to two different types of novel environments following recording of EEG in the home cage: the Novel Open Field (NOF, for details see [72]) and the Elevated Plus Maze (EPM, for details see [72]). We used the first 30s of exploration in these novel environments for calculations of CFC and compared those to CFC during 30s of previous spontaneous exploration in the home cage. CFC during REM-sleep was based on collected EEG in the home cage before and after the exposure to novel environments.

### Data Analysis

Continuous 10 h EEG recordings either at the circadian active phase (from 10:00 to 20:00) or at the quiet phase (from 21:00 to 7:00) of the ten male animals were imported into a MATLAB-based program (The Mathworks Inc., Natick, MA) using built-in and custom written routines. The neck muscle EMG activity was high pass filtered (30 Hz). Visual classification of REM-sleep and active wakefulness (aWk) was based on: 1) the level of EMG activity (aWk > REM); 2) the amount of high amplitude-low frequency delta activity in the neocortex (NREM > quiet waking, aWk and REM; 3) the amount of regular theta oscillations in the lateral parietal cortex overlaying the dorsal hippocampus (REM and aWk > quiet waking and NREM); 4) neck muscle activity (REM<NREM<qWk<aWk). For detailed description of behavioural staging see [35]. To measure the intensity of phase-amplitude coupling we used the modulation index (MI) described in detail in [40]. The MI is capable of assessing coupling between two frequency ranges of interest: a slower phase-modulating (f_p_) and a faster amplitude-modulated (f_a_) frequency. Briefly, the MI is computed as follows: the phases of f_p_ are binned into eighteen 20° intervals and the mean amplitude of f_a_ in each phase bin is determined. The mean f_a_ amplitude in each phase bin is normalized so that its sum over all phase bins equals 1. This gives rise to a phase-amplitude “distribution” (see Fig. 5A,B for examples). A uniform phase-amplitude distribution means that the f_a_ amplitude is on average the same for all f_p_ phases, which happens in the absence of phase-amplitude coupling. The higher the coupling, the further away the phase-amplitude distribution gets from the uniform distribution. The MI is essentially a measure of divergence of the phase-amplitude distribution from the uniform distribution, normalized to achieve values between 0 and 1 [40]. The comodulation map (see Fig. 3B) is obtained by computing the MI of several frequency band pairs and expressing the results in a two-dimensional pseudocolor plot. One dimension denotes the frequency bands analyzed as phase-modulating and the other dimension represents the amplitude-modulated bands. The frequency bands are narrow-filtered (phase frequencies: 4-Hz bandwidths; amplitude frequencies: 10-Hz bandwidths) and each coordinate in the comodulogram represents the center frequency. A warm color in a (f_p,_f_a_) entry of the comodulogram means that the phase of f_p_ modulates the amplitude of f_a_. Of note, we have employed some techniques described in [73] to rule out that the CFC effects we observed are artifacts of the shape of the theta wave (see Text S2).

The time-frequency amplitude distribution time-locked to the theta peak shown in Figure 5C was obtained by first localizing the time points corresponding to the theta peaks, and then computing the mean amplitude envelope centered at these time points; this procedure was performed for several narrow filtered frequency bands of 4-Hz bandwidth, covering 10 to 200 Hz with 2-Hz steps. In order to control for 1/f, the amplitude of oscillations at different frequencies was normalized by dividing the amplitude values at each time point by the mean amplitude.

Power spectral density (PSD) functions (FFT, Hanning-window, window size: 4s, step: 2s for data shown in Fig. 3; window size 1s, step: 0.5s for data shown in Fig. 2 and 4) were used to calculate band power (i.e. mean power of all frequencies within the band) and power maximum (i.e. power of the dominant frequency) for three frequency bands: theta (4–12 Hz), gamma (40–100 Hz), and fast gamma oscillations (120–160 Hz).

Analysis of possible interactions between CFC and theta power (Fig. 4) required normalization of theta power for each animal. This was achieved in three steps: 1) total power between 1 and 200 Hz was computed for each 1s data epoch; 2) the median of total power from all those epochs was calculated; 3) theta power between 4 and 12 Hz was divided by the median of total power. We then pooled 30s of data from each animal into bins of 0.01 values of normalized theta power and averaged across animals. For non normalized theta power (Fig. 4A,C) 30s of data from each animal were pooled into bins of 50 power values. To allow comparing the power levels of multiple frequency bands in the same scale, the PSD in Figures 2C,D and 3A are expressed as dB relative to unity.

### Statistics

Data are expressed as means and standard error of the means (SEM). For group comparisons of normally distributed data (Kolmogorov-Smirnov test) we used the paired t-test. For data with non-Gaussian distribution we used the nonparametric test of Wilcoxon matched pairs test. Different significance levels are shown in figures with one to three asterisks (*: p<0.05, **: p<0.005, ***: p<0.0005).

## Supporting Information

Figure S1 **Fast gamma and gamma oscillations are present in raw field potentials.** A: Raw field potential traces from parietal cortex during REM-sleep. Gamma-filtered (red curve) and fast gamma-filtered (blue curve) signals are also shown. Notice that both oscillations can be observed in the unfiltered signal. B: Averaging unfiltered field potentials triggered by the peak of the gamma (upper trace) or fast gamma (lower trace) waves reveals gamma and fast gamma oscillations nested in the theta wave.
(TIF)

Figure S2 **Fast gamma oscillations are more burst-like than gamma both in active waking (aWk) and REM-sleep (REM), but changes in bursting activity do not account for increased theta-fast gamma coupling during REM.** Mean coefficient of variation (CV) of the instantaneous amplitude of fast gamma and gamma oscillations is shown. Higher CV values indicate higher variation from the background mean, or “burstiness”. Notice that fast gamma oscillations during REM-sleep are not more “bursting” than fast gamma oscillations in aWk. Therefore, according to this analysis, “burstiness” can not explain the CFC differences between REM and aWk.
(TIF)

Figure S3 **State-dependent changes in burst-like behaviour of fast gamma and gamma oscillations (second set of analyses).** A: Instantaneous amplitude distribution for gamma (red) and fast gamma (blue) oscillations during aWk (left) and REM (right) states. For comparison between the two frequency bands, the amplitude values were z-score normalized (that is, 0 denotes the mean amplitude, and the x-axis represents the number of standard deviations above (+) or below (−) the mean amplitude). The inset plots show the distribution of high amplitude values, which characterize bursting activity. Notice similar amplitude distributions between aWk and REM. In particular, notice in the inset plots that fast gamma oscillations have higher probability of high amplitude values in both aWk and REM, indicating that fast gamma oscillations are more bursting than gamma in both these states. B: Same results as in the two inset plots above, but reproduced in the same panel to allow direct comparison. Notice that fast gamma oscillations in REM have lower probability of showing high amplitude values than during aWk. C: Number of amplitude threshold crossings per second for gamma (left bars) and fast gamma (right bars) oscillations during REM and aWk. The threshold was chosen as five standard-deviations (SD) above the background mean. According to this criterion, notice that fast gamma oscillations are more bursting than gamma, and that the level of “burstiness” cannot explain the CFC differences between REM and aWk states.
(TIF)

Figure S4 **Representative example of cross-regional coupling during REM-sleep between parietal cortex and CA1.** A: Raw and filtered (100–200 Hz) traces in parietal cortex and CA1 (below pyramidal cell layer). Note the 180° phase shift of theta waves in CA1 compared to neocortex (dotted line). B: Comodulogram maps of 30s periods of REM. Note that theta-fast gamma CFC is restricted to neocortex, whereas theta-gamma CFC dominates in CA1.
(TIF)

Figure S5 **Cross-regional coherence and frequency plots during REM (n = 10 mice, means and S.E.M., 180s REM each).** A: Coherence spectrum between parietal cortex (par cx) and deep CA1 (below pyramidal cell layer) shows lowest coherence values in the fast gamma frequency range. B: Coherence between parietal cortex and surface CA1 (surf CA1, above pyramidal cell layer) also shows low coherence in the fast gamma frequency range. Arrows indicate theta, gamma and fast gamma peaks. High coherence values support volume conduction, as is the case of theta oscillations, whereas low coherence suggests lack of volume conduction.
(TIF)

Figure S6 **Exposure to novel environments has no effect on theta-fast gamma or theta-gamma CFC in the parietal cortex, neither during exploration (aWk) nor in REM-sleep prior or after the exposure.** A: Mean comodulation maps (CFC) during 30s spontaneous active waking (spont aWk) in the home cage compared to first 30s in a Novel Open Field (NOF); B: Mean CFC during 30s of REM-sleep prior to NOF compared to 30s REM immediately after NOF. REM-sleep periods were recorded in the home cage; C,D: Similar results as in A and B but for mice in a Elevated Plus Maze (EPM) (A,B,C,D: n = 9 mice).
(TIF)

Text S1 **“Burstiness” as a confounding factor.**
(DOC)

Text S2 **Sharp edges as a confounding factor.**
(DOC)
