# Supplementary figures and images for: Selective Coupling between Theta Phase and Neocortical Fast Gamma Oscillations during REM-Sleep in Mice
Source: PLoS One. 2011 Dec 5;6(12):e28489. doi: 10.1371/journal.pone.0028489 (PMC3230633; doi:10.1371/journal.pone.0028489)

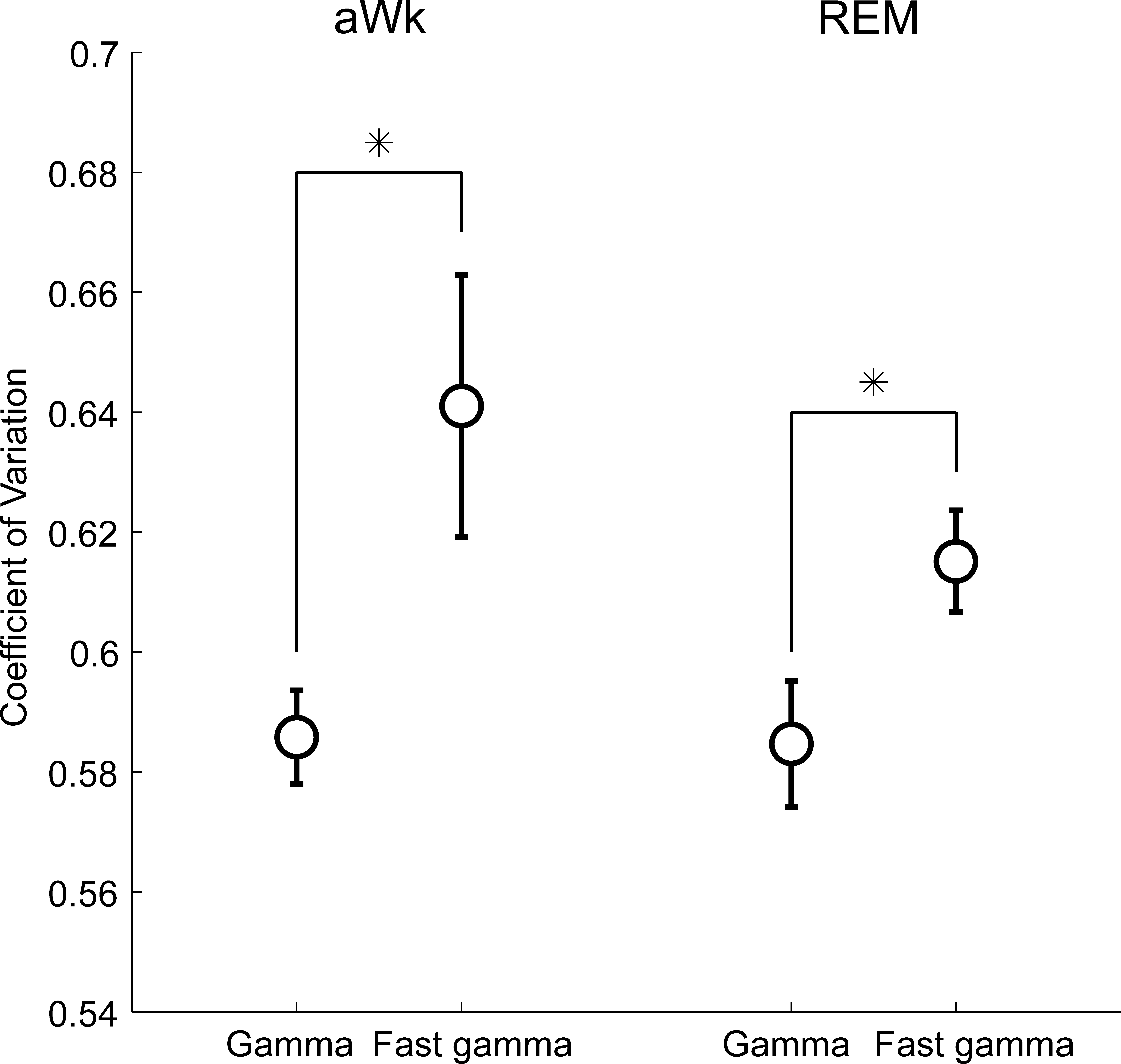

Supplement: Figure S2 — Fast gamma oscillations are more burst-like than gamma both in active waking (aWk) and REM-sleep (REM), but changes in bursting activity do not account for increased theta-fast gamma coupling during REM. Mean coefficient of variation (CV) of the instantaneous amplitude of fast gamma and gamma oscillations is shown. Higher CV values indicate higher variation from the background mean, or “burstiness”. Notice that fast gamma oscillations during REM-sleep are not more “bursting” than fast gamma oscillations in aWk. Therefore, according to this analysis, “burstiness” can not explain the CFC differences between REM and aWk. (TIF) [file pone.0028489.s002.tif]

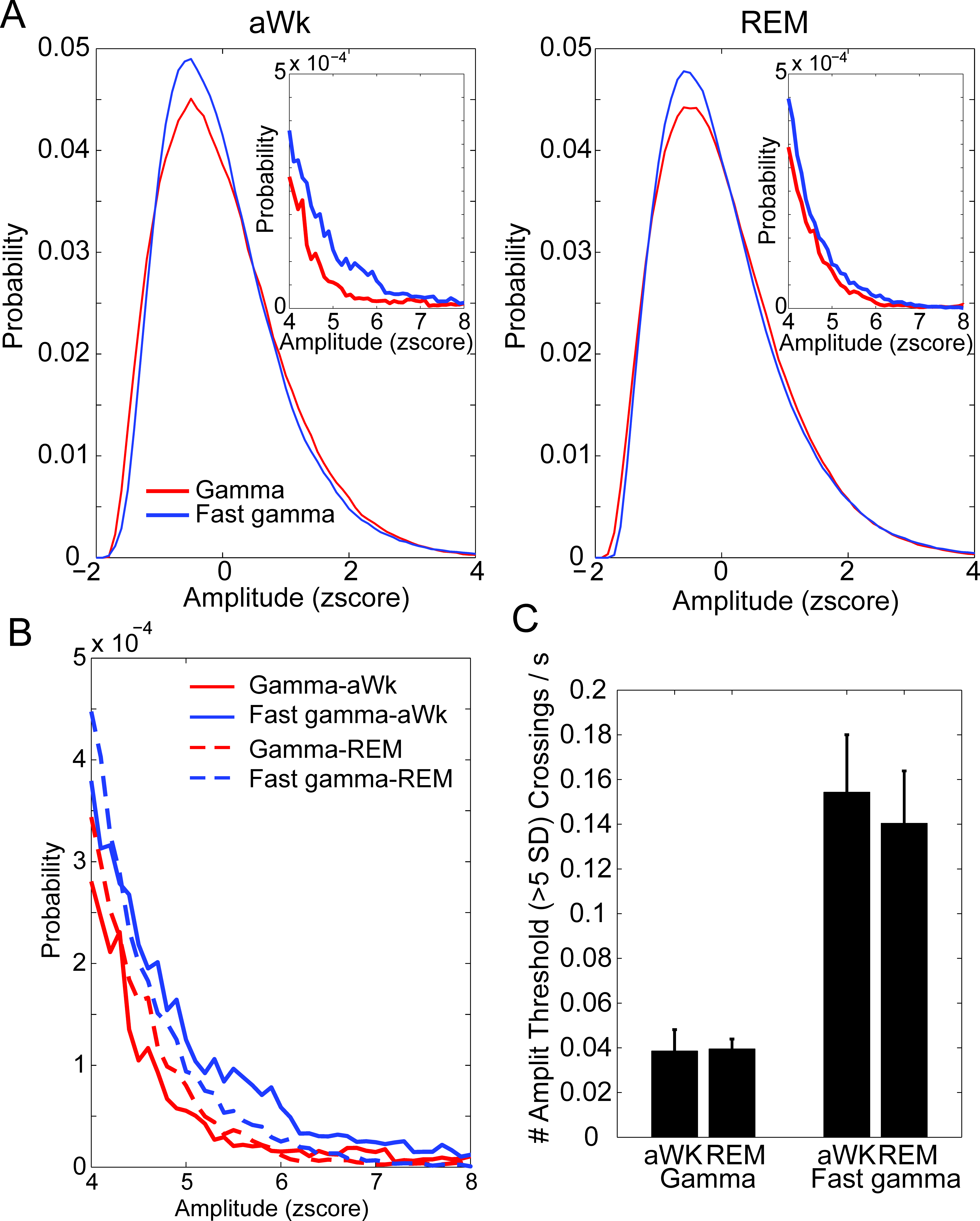

Supplement: Figure S3 — State-dependent changes in burst-like behaviour of fast gamma and gamma oscillations (second set of analyses). A: Instantaneous amplitude distribution for gamma (red) and fast gamma (blue) oscillations during aWk (left) and REM (right) states. For comparison between the two frequency bands, the amplitude values were z-score normalized (that is, 0 denotes the mean amplitude, and the x-axis represents the number of standard deviations above (+) or below (−) the mean amplitude). The inset plots show the distribution of high amplitude values, which characterize bursting activity. Notice similar amplitude distributions between aWk and REM. In particular, notice in the inset plots that fast gamma oscillations have higher probability of high amplitude values in both aWk and REM, indicating that fast gamma oscillations are more bursting than gamma in both these states. B: Same results as in the two inset plots above, but reproduced in the same panel to allow direct comparison. Notice that fast gamma oscillations in REM have lower probability of showing high amplitude values than during aWk. C: Number of amplitude threshold crossings per second for gamma (left bars) and fast gamma (right bars) oscillations during REM and aWk. The threshold was chosen as five standard-deviations (SD) above the background mean. According to this criterion, notice that fast gamma oscillations are more bursting than gamma, and that the level of “burstiness” cannot explain the CFC differences between REM and aWk states. (TIF) [file pone.0028489.s003.tif]

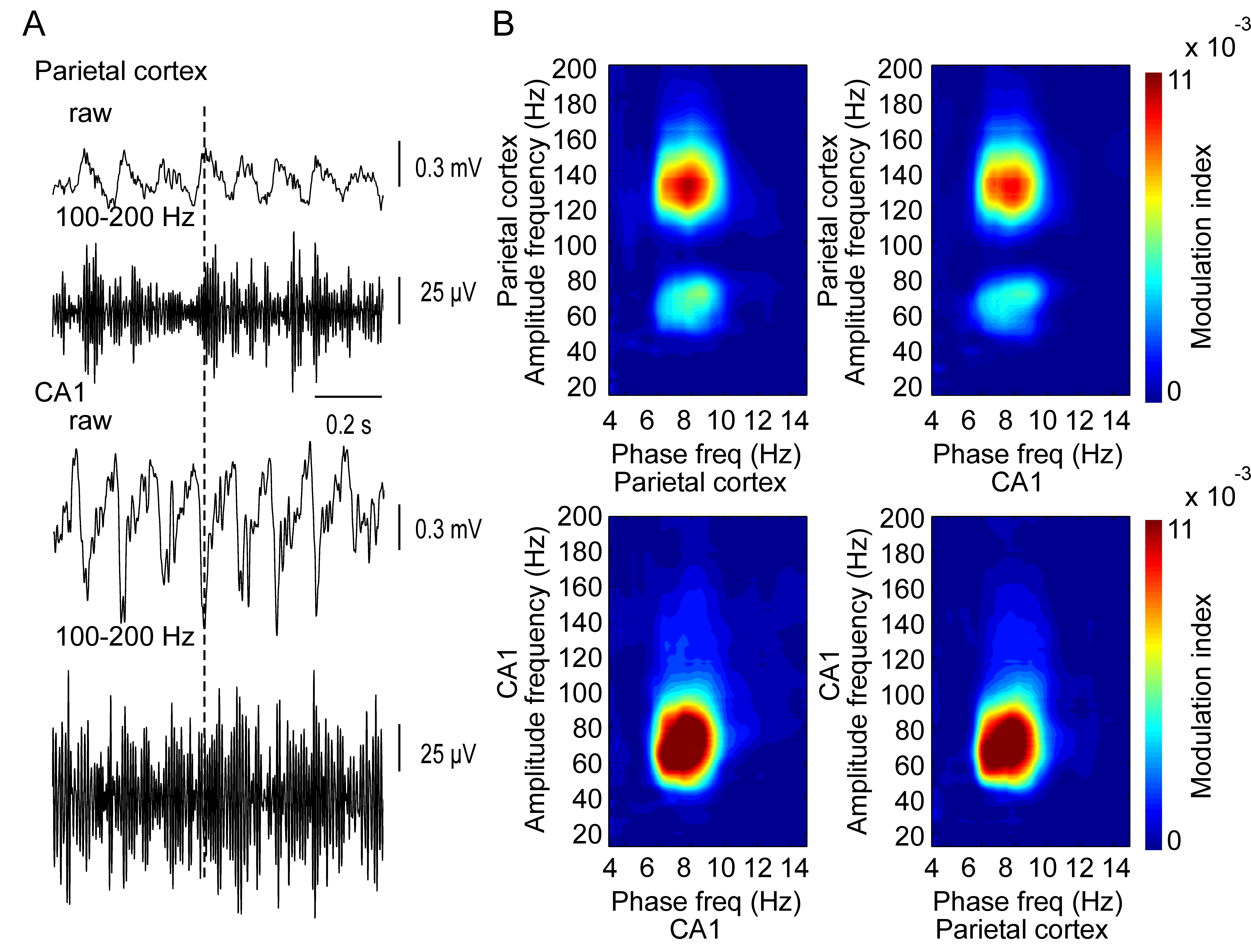

Supplement: Figure S4 — Representative example of cross-regional coupling during REM-sleep between parietal cortex and CA1. A: Raw and filtered (100–200 Hz) traces in parietal cortex and CA1 (below pyramidal cell layer). Note the 180° phase shift of theta waves in CA1 compared to neocortex (dotted line). B: Comodulogram maps of 30s periods of REM. Note that theta-fast gamma CFC is restricted to neocortex, whereas theta-gamma CFC dominates in CA1. (TIF) [file pone.0028489.s004.tif]

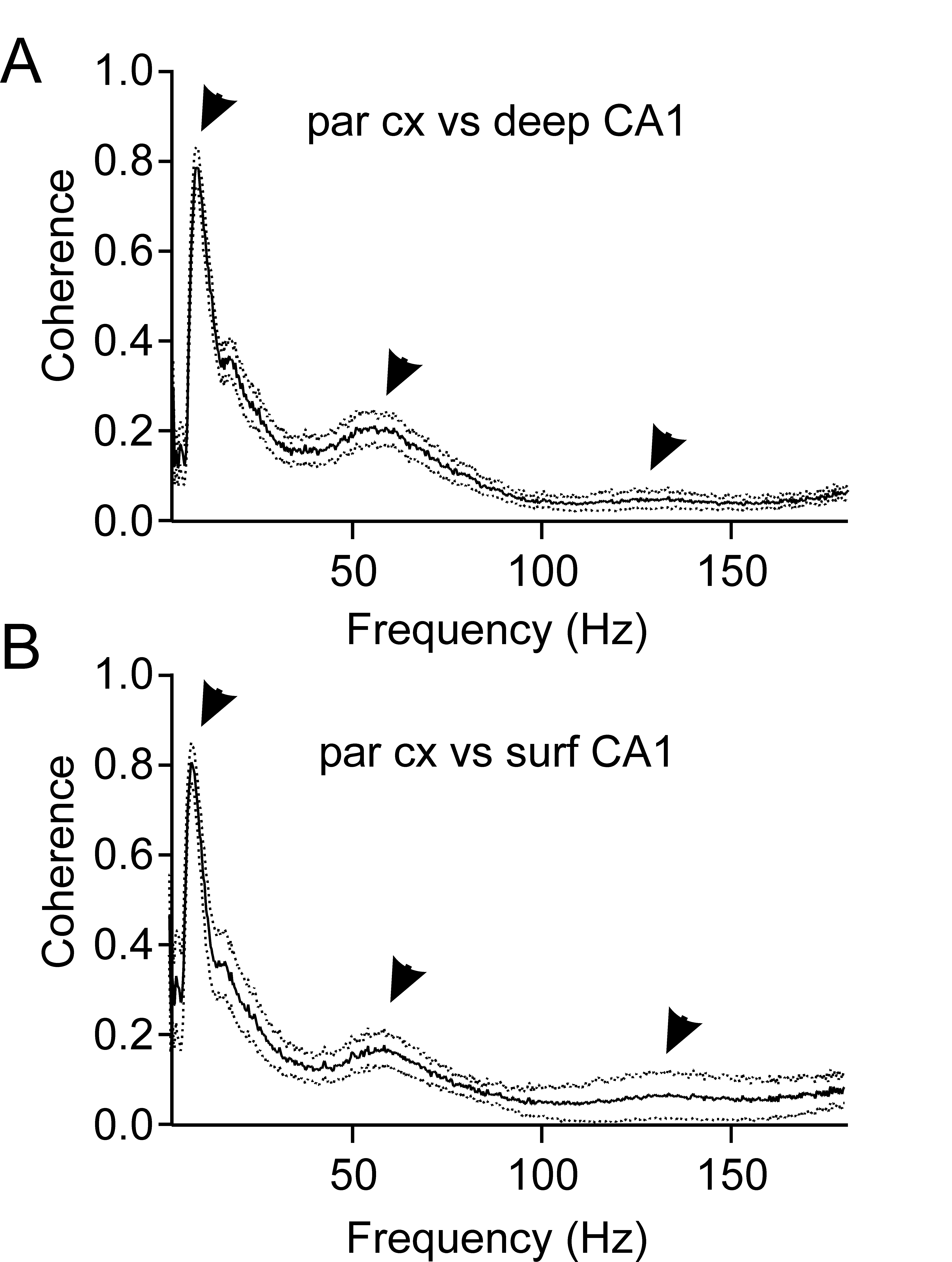

Supplement: Figure S5 — Cross-regional coherence and frequency plots during REM (n = 10 mice, means and S.E.M., 180s REM each). A: Coherence spectrum between parietal cortex (par cx) and deep CA1 (below pyramidal cell layer) shows lowest coherence values in the fast gamma frequency range. B: Coherence between parietal cortex and surface CA1 (surf CA1, above pyramidal cell layer) also shows low coherence in the fast gamma frequency range. Arrows indicate theta, gamma and fast gamma peaks. High coherence values support volume conduction, as is the case of theta oscillations, whereas low coherence suggests lack of volume conduction. (TIF) [file pone.0028489.s005.tif]

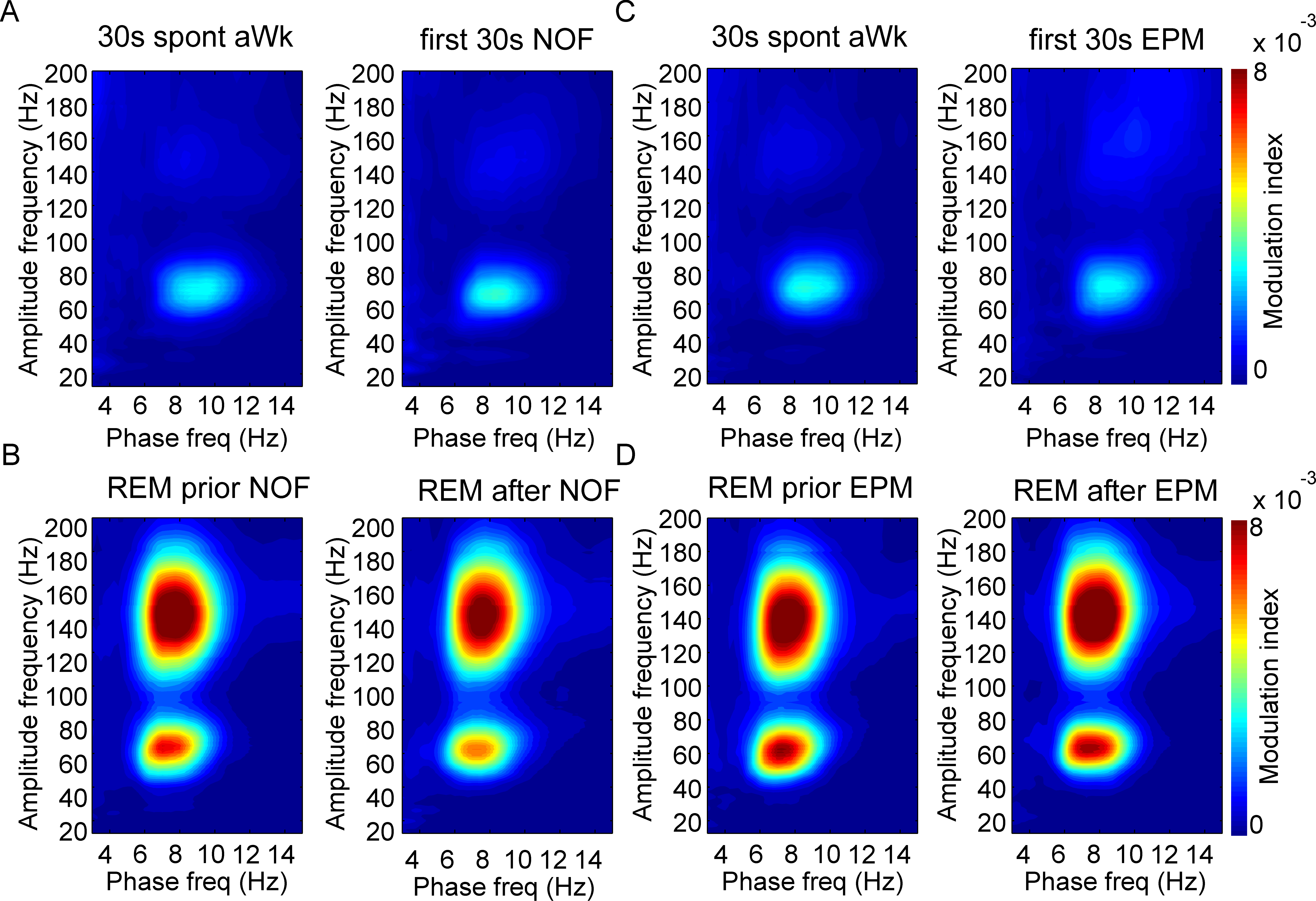

Supplement: Figure S6 — Exposure to novel environments has no effect on theta-fast gamma or theta-gamma CFC in the parietal cortex, neither during exploration (aWk) nor in REM-sleep prior or after the exposure. A: Mean comodulation maps (CFC) during 30s spontaneous active waking (spont aWk) in the home cage compared to first 30s in a Novel Open Field (NOF); B: Mean CFC during 30s of REM-sleep prior to NOF compared to 30s REM immediately after NOF. REM-sleep periods were recorded in the home cage; C,D: Similar results as in A and B but for mice in a Elevated Plus Maze (EPM) (A,B,C,D: n = 9 mice). (TIF) [file pone.0028489.s006.tif]
