# Supplementary material for: Selective Coupling between Theta Phase and Neocortical Fast Gamma Oscillations during REM-Sleep in Mice
Source: PLoS One. 2011 Dec 5;6(12):e28489. doi: 10.1371/journal.pone.0028489 (PMC3230633; doi:10.1371/journal.pone.0028489)
Supplement: Text S1 — “Burstiness” as a confounding factor. (DOC) [file pone.0028489.s007.doc]

**Supporting Text 1 – “Burstiness” as a confounding factor**

A possible cause for the observed difference of theta-gamma and theta-fast gamma coupling between active wakefulness (aWk) and REM-sleep (REM) could be a difference in “burst-like” oscillations (“burstiness”), i.e. in the time-dependent variation of the amplitude of each oscillation. To investigate this possibility we adopted three approaches. First, we computed the coefficient of variation of the amplitude envelope of gamma and fast gamma oscillations during aWk and REM. A “burst-like” oscillation should result in a larger coefficient of variation of its amplitude envelope. According to this metric, we found that fast gamma oscillations have a greater variation of their amplitude envelope than gamma oscillations. However, the coefficient of variation of fast gamma oscillations during REM sleep was not larger than in aWk, as shown in the Figure S2. Therefore, coefficient of variation cannot explain the increase in theta-fast gamma CFC during REM.

Second, we analyzed the distribution of the amplitude envelope in units of standard deviations (SD) from the mean amplitude (that is, we studied the distribution of the z-scored amplitude). We reasoned that a greater level of burst activity would be characterized by a greater number of amplitude values above a certain threshold (say, 5 SD). Again, we found that fast gamma oscillations have a greater “burstiness” than gamma oscillations, as shown in the insets of the Figure S3A. However, using this analysis, we again did not find more “burstiness” in REM compared to aWk (Figure S3B). Third, we investigated the number of times at which the amplitude envelope crossed a given threshold (from below) per unit of time. We chose a threshold of 5 SD above the mean amplitude (other threshold values provide similar results). As before, we found no increased “burstiness” in REM compared to aWK (Figure S3C). We therefore conclude that an increase in the “burst-like” activity of fast gamma oscillations is unlikely to explain our CFC results.
