# Supplementary material for: Selective Coupling between Theta Phase and Neocortical Fast Gamma Oscillations during REM-Sleep in Mice
Source: PLoS One. 2011 Dec 5;6(12):e28489. doi: 10.1371/journal.pone.0028489 (PMC3230633; doi:10.1371/journal.pone.0028489)
Supplement: Text S2 — Sharp edges as a confounding factor. (DOC) [file pone.0028489.s008.doc]

**Supporting Text 2 – Sharp edges as a confounding factor**

As detailed in Kramer and colleagues [1] there are a couple of ways to test whether sharp edge effects cause spurious high frequency oscillations. A first simple but important procedure is visual inspection of raw traces. Is the oscillation visible in the unfiltered signal? Does it originate from sharp deflections of the theta wave? In our case, we observed that fast gamma and gamma are genuine oscillations that co-occur with theta waves in the unfiltered local field potentials (LFP, Figure S1A). The direct observation in the LFP (which did not undergo band pass filtering) excludes that the fast oscillations are filtering artefacts. Secondly, both types of fast oscillations can be observed in plots of power spectral density (PSD) as shown in Figure 3A. This does indicate that oscillations in the gamma range and in the range of ~ 120-150 Hz (fast gamma) exist as genuine network activity. High-frequency harmonics can, in principle, result from deviations of the theta waves from a pure sinusoidal wave form. However, these oscillations would express decreasing peaks at multiples of the theta wave frequency (i.e., 8, 16, 24, 32, 40 Hz etc). Lastly, as pointed out in Kramer et al. 2008 [1], spurious coupling can be distinguished from genuine oscillations by averaging the unfiltered field potential triggered by the peaks of the high frequency activity. If oscillating field potentials result from sharp edge artefacts, this procedure does not lead to visible oscillations in the averaged trace. Genuine theta-nested oscillations, on the other hand, should yield oscillations in the averaged trace (this effect is illustrated in the upper left and right panels of Fig. 4 in [1]). Applying this technique to our data, we found the averaged traces shown in Fig. S1B (upper trace: gamma peak-triggered average; bottom trace: fast gamma-peak-triggered average). These findings strongly speak against sharp-edge artefacts. It should also be noted that theta oscillations are much sharper and much larger in amplitude in CA1 below the pyramidal cell layer as compared to the neocortex (see reference [2] and raw traces in Fig. S4A). Nevertheless, we find no prominent coupling between theta and fast gamma oscillations in this region (see Fig. S4B and [3]).

References

1. Kramer MA, Tort ABL, Kopell NJ. (2008) Sharp edge artifacts and spurious coupling in EEG frequency comodulation measures. J Neurosci Methods. 170: 352-357.
2. Buzsáki G, Czopf J, Kondákor I, Kellényi L. (1986) Laminar distribution of hippocampal slow activity (RSA) in the behaving rat: Current-source density analysis, effects of urethane and atropine. Brain Res. 365: 125-137.
3. Scheffer-Teixeira R, Belchior H, Caixeta FV, Souza BC, Ribeiro ST, et al. (2011) Theta phase modulates multiple layer-specific oscillations in the CA1 region. Cerebral Cortex, In press.
